# The effect of peer-led education on the life quality of mastectomy patients referred to breast cancer-clinics in Shiraz, Iran 2009

**DOI:** 10.1186/1477-7525-8-74

**Published:** 2010-07-23

**Authors:** Farkhondeh Sharif, Narjes Abshorshori, Sedigheh Tahmasebi, Maryam Hazrati, Najaf Zare, Sarah Masoumi

**Affiliations:** 1 Mental Health Nursing Department, Fatemeh (P.B.U.H) Faculty of Nursing and Midwifery, Shiraz University of Medical Sciences, shiraz-Iran; 2 Nursing Department, Faculty of Nursing affiliated to Shiraz University of Medical Sciences, Larestan - Iran; 3 Department of Surgery, Shiraz University of Medical Sciences, Shiraz-Iran; 4 Medical Surgical Nursing Department, Fatemeh (P.B.U.H) Faculty of Nursing and Midwifery, Shiraz University of Medical Sciences, Shiraz, Iran; 5 Shiraz University of Medical Sciences, Department of Biostatistics, Faculty of Medicine, Shiraz-Iran; 6 Iranian Journal of Medical Sciences Office, Shiraz University of Medical Sciences, Shiraz-Iran

## Abstract

**Background:**

Breast cancer among women is a relatively common with a more favorable expected survival rates than other forms of cancers. This study aimed to determine the improved quality of life for post-mastectomy women through peer education.

**Methods:**

Using pre and post test follow up and control design approach, 99 women with stage I and II of breast cancer diagnosis were followed one year after modified radical mastectomy. To measure the quality of life an instrument designed by the European organization for research and treatment of cancer, known as the Quality of Life Question (QLQ-30) and it's breast cancer supplementary measure (QLQ-BR23) at three points in time (before, immediately and two months after intervention) for both groups were used. The participant selection was a convenient sampling method and women were randomly assigned into two experimental and control groups. The experimental group was randomly assigned to five groups and peer educators conducted weekly educational programs for one month. Tabulated data were analyzed using chi square, t test, and repeated measurement multivariate to compare the quality of life differences over time.

**Results:**

For the experimental group, the results showed statistically significant improvement in all performance aspects of life quality and symptom reduction (P < 0.001), while the control group had no significant differences in all aspects of life quality.

**Conclusion:**

The findings of this study suggest that peer led education is a useful intervention for post-mastectomy women to improves their quality of life.

## Background

Breast cancer can be a life threatening disease for women worldwide [1]. In Iran breast cancer consists of 22.6% of all cancers affecting women [2]. Most women diagnosed with breast cancer are between 35 to 44 years of age and this rate drops after the age of 44 which is 10 years less than the western figures for women with breast cancer [3,4]. The study by Mehrabani et al. (2008) on cancer occurrence in the Fars Province (southern Iran) indicated breast cancer as one of the top 10 cancers among women in a 5-years study of registered patients [5]. Surgery, chemotherapy, radiotherapy and hormone therapy are the most common methods for breast cancer treatment in Iran which have lengthened the survival rate of these people. Although treatments have shown some survival rate success, their negative impacts on the quality of life are under reported. Psychological distress, anxiety and depression were found to be common among breast cancer patient's even years after the diagnosis of the disease and treatment (6). In a survey by Hack & Degner on 1249 women newly diagnosed with breast cancer, the results showed that 32.8% had experienced psychological distress [7].The loss of one or both breasts evoked feelings of mutilation and altered body image, diminished self-worth, loss of a sense of feminity, reduction of sexual attractiveness and function, anxiety, depression, hopelessness, guilt, shame and fear of recurrence and death [8]. Also Siberfarb and his colleagues compared the psychosocial status in groups of breast cancer patients during initial diagnosis (N= 50), first recurrence (N = 52), and metastatic disease (N = 44). Their findings indicated that the stage of first recurrence was the most emotionally stressful time in their samples [9]. In this regard Lewis & Deal in their study of 15 married couples in where the wife was diagnosed with a recurrence of breast cancer found problems in marital adjustment as well as depression among 40% of the women [10]. Besides these symptoms the patients suffered from pain syndrome, lymphocyte edema, shoulder movement restriction, and muscle atrophy[11]. Dawes et al, in a study on 204 post-mastectomy patients found that 35% of them had one or more symptoms of lymph edema and they found a significant relationship between pain and activity limitation and participation restriction [12]. A variety of intervention types such as psychological, behavioral and formats such as group, individual and telephone have demonstrated beneficial effect on the quality of life, symptom management and psychological functioning [13]. Visiting the same patients with the same diagnosis would bring relief and assurance for patients to overcome the disease, leading to a higher life expectancy. When women who have breast cancer are in touch with each other, they have empathy towards each other and they would widely discuss their experiences and difficulties creating a supportive environment for transfer of knowledge and awareness [14]. It has been reported in an observational study on the effect of peer counseling on the quality of life following diagnosis of breast cancer that women expressed the greatest need for counseling and they wanted to speak with someone who had the same disease and had gone through the crisis of treatment and is leading a normal life [15]. Considering an increase in the number of breast cancer patients and limitation in the physicians time to educate them, this study highlights the importance of peer-lead education on the life quality after mastectomy for women who experienced breast cancer and recruited from breast cancer clinics in Shiraz, southern Iran.

## Methods

### Design

This is an intervention, pre-post test follow up and control group design study aiming to examine the effect of peer-lead education on the life quality of women post-mastectomy. The convenience and a purposeful sampling method helped randomly divide the participants into two experimental and control groups. All the patients met the inclusion criteria as stage I or II breast cancer diagnosis, having had modified radical mastectomy, at least 1 years after their mastectomy, completed chemotherapy and radiotherapy, and currently under hormone therapy. Exclusion criteria were having other types of malignancy or psychiatric disorder. To obtain sufficient statistical power in detecting differences between the groups and to predict the study outcomes between the two groups with significance of (p < 0.05), one hundred women were selected. They were randomly assigned into experimental and control groups. Of the experimental group one person discontinued leaving 49 patients in the experimental group and 50 in the control group.

The quality of life was measured using the European organization for research and treatment of cancer quality of life question (QLQ-30) and its breast cancer supplementary measure (QLQ-BR23) at three points in time: before, immediately, and two months after the intervention in both groups. The instrument was administered blindly by the researcher. The instrument developed by the European Organization for research on the quality of life for cancer patients (EORTC QLQ-30) consists of functional and symptomatic scales. The functional scales consist of the general health condition, physical function, mental function, psychological function and the social function. The symptomatic scales consists of fatigue, sickness and vomiting, pain, dispend, insomnia, loss of appetite, constipation, diarrhea and economical problems, each one consisting of a number of questions. The questionnaire by the European organization for research and treatment of cancer (QLQ-BR23) is merely specialized for breast cancer and measures the life quality of patients suffering from breast cancer. This questionnaire evaluates four functional aspects and four sets of common symptoms in patients suffering from cancer. It consists of mental conception of the body, sexual function, sexual function satisfaction, and attitude towards future, side effects of systemic treatments, side effects in the patient's breast and hand and worries about hair loss. The score of each aspect is based on a scale of 0 to 100. In functional and general aspects of life quality a higher score shows a better functional condition or a better life quality whereas, in the symptoms aspect, a high score is a sign of problem. Validity and reliability of its Persian (Farsi) script was assessed by Montazeri et. al. (1999) in Iran and it was introduced as a valid and reliable tool by the European organization for research and treatment of cancer [16,17]. The collected data were analyzed by SPSS software, Chi-square tests, independent T-test and the repeated measurement multivariate tests for measuring life quality in three different time periods.

The experimental group was divided into five subgroups of ten patients and carried on in two stages. In the first stage and based on the patient's physician's opinion 5 of the patients who were in stages I and II of the illness with at least five years remission post-mastectomy and able to communicate with others were selected as the peer educators. Their training was about the concept of cancer, breast cancer, diagnosis, treatment, complications, self care, relaxation techniques and adaptation to the illness. The training was performed in 4 sessions and each session lasted for one hour. The training sessions for peer educators were conducted by experts in the psycho-oncology field. In the second stage these peer educators were asked to go to the groups and after needs assessment, guide the group in a friendly environment. For each group peer educators conducted 4 sessions on a weekly basis for one month. Each session lasted approximately 1 hour and started with refreshment and continued with discussion about the topics according to the group's need. The control group did not receive any intervention and after the last stage of data collection they received an educational pamphlet.

Institutional Review Board (IRB) approval for the study was obtained from the Ethics Committee of Shiraz University of Medical Sciences (ECSUMS). Written consent was obtained from each patient. The purpose of study, voluntary participation, confidentiality and freedom to discontinue at any time was reviewed. The study was carried out in the only breast cancer clinic in Shiraz in Fars province at southern Iran. This is the largest clinic in Fars Province which is affiliated with Shiraz University of Medical Sciences. This center offers services (treatment and follow up) to at least 50 patients on a daily basis.

## Results

The collected data were analyzed using SPSS software, Chi-square tests, independent T-test and the repeated measurement multivariate tests for assessing life quality in three different time periods. Table 1, presents the demographic and baseline characteristics of the experimental and control groups. The results revealed that the experimental and control groups were similar with respect to age, marital status and educational level of the patients. The majority of the participants were aged between 40 to 49 years (32.7% in the experimental group and 40% in the control group), and most of them were married (88.8% in the experimental group and 90% in the control group). There was no significant difference in the demographic variables between the two groups.

**Table 1:** Demographic Characteristics of the study sample in groups

	No (%) Experimental Group	No (%) Control Group	p
Age Groups (Years)			
30-39	10(20.4)	7(14)	
40-49	16(32.7)	20 (40)	0.810
50-59	14(28.6)	14(28)	
60-70	9(18.4)	9 (18)	
			
Educational Level			
Below Diploma	27(55.1)	26(52)	
Above Diploma	22(44.9)	26(52)	0.528
			
Marital Status			
Single	3(6.1)	2(4)	
Married	43(88.8)	45(90)	0.889
Divorced or widow	3(6.1)	3(6)	
			
Total	49 (100)	50 (100)	

Assessing the results before interventions with the independent T-test showed that the two groups were similar in almost all of the functional and symptomatic scales of quality of life regarding cancer and breast cancer and there was no statistically significant difference between the two groups before intervention.

In comparing the patients' functioning and global life quality before and after intervention which was measured by the EORTC QLQ-C30 the results while considering the mutual effect of time and group indicated significant improvement in the experimental group and a reduction in the control group's function. Time was a significant factor for change in the experimental and control groups in aspects such as general health condition, psychological function, and social function, mental function of life quality regarding cancer and the body image, sexual function, and satisfaction with the sexual function regarding the breast cancer patients' life quality. However, in general, regardless of time there was no significant difference between the experimental and control groups (P = 0.208). No specific difference was seen in the physical function before to two months after intervention and thus it seems that time was not a significant factor for change in this aspect (P = 0.777). The experimental group's function accelerated and the control group's function decreased (P = 0.041) (Table 2)

**Table 2:** Functioning and Global quality of life mean scores in groups before and after the intervention (as measured by the EORTC QLQ-C30)

Functioning Scores	Time	Before intervention	After intervention	2 months after intervention	Time	Group	Time/group
			
	Groups	Mean ± SD	Mean ± SD	Mean ± SD			P
Global health status	Case	63.09 ± 22.69	68.19 ± 19.06	80.0 ± 17.90	*	*	*
							
	Control	62.16 ± 22.02	61.83 ± 22.02	61.66 ± 21.88	.001	.o37	.001

Physical functioning	Case	79.72 ± 18.55	80.54 ± 17.83	80.81 ± 16.02	.777	.425	*
							
	Control	83.26 ± 13.41	82.58 ± 13.37	82.27 ± 13.32			.041

Role functioning	Case	85.37 ± 21.95	87.75 ± 18.24	98.63 ± 5.72	*		*
							
	Control	86.80 ± 19.73	86.45 ± 20.23	85.06 ± 20.69	.001	.208	.001

Cognitive functioning	Case	72.78 ± 23.24	85.71 ± 15.21	97.75 ± 5.52	*	*	*
							
	Control	70.74 ± 23.20	70.40 ± 23.14	69.38 ± 24.13	.001	.001	.001

Emotional functioning	Case	63.94 ± 29.23	85.37 ± 13.45	97.10 ± 4.97	*	*	*
							
	Control	61.90 ± 25.45	60.71 ± 24.65	60.20 ± 24.73	.001	.001	.001

Social functioning	Case	81.97 ± 18.89	91.15 ± 13.22	99.65 ± 2.38	*	*	*
							
	Control	81.97 ± 20.92	80.27 ± 20.032	78.57 ± 22.30	.001	.001	.001

The higher value indicate higher level of functioning and quality of life, min: 0, max: 100

Comparison of the patients' symptom, the mean score from pre to post and follow up intervention indicated the change process regarding fatigue and insomnia from the quality of life in relation to cancer. Also, the side effects of systemic treatments in the control group differed from the quality of life in relation to cancer. Exactly after the intervention and two months after it the symptomatic scales indicated a decline in 4 aspects including fatigue, Pain, insomnia and loss of appetite (it is shown in Table 3 with *) in the experimental group and an increased in the control group. Time was a significant factor for change in these aspects and these changes were more evident in the experimental group in decreasing the symptoms (P < 0.001). Generally, regardless of time there was no significant difference between the experimental and control groups regarding fatigue (P = 0.149), insomnia (P = 0.547) and the side effects of systemic treatments. Regarding aspects such as nausea and vomiting (P = 0.320), constipation (P = 0.076), economical condition (P = 0.608) and dyspnea (P = 0.167), time was not a significant factor for change (Table 3).

**Table 3:** Means scores of Symptoms of life quality in groups before and after the intervention (as measured by the EORTC QLQ-C30)

Symptom Scores	Time	Before intervention	After intervention	2 months after intervention	Time	Group	Time/group
			
	Groups	Mean ± SD	Mean ± SD	Mean ± SD			P
Fatigue	Case	25.62 ± 19.00	19.04 ± 15.87	8.61 ± 12.88	*	.149	
							
	Control	22.44 ± 21.35	23.11 ± 21.62	23.77 ± 21.99	.001		.001

Nausea and vomiting	Case	20.76 ± 18.36	19.95 ± 17.68	17.00 ± 19.09	*		
							
	Control	12.33 ± 20.42	11.00 ± 18.93	11.33 ± 19.17	0.297	.116	.320

Pain	Case	21.42 ± 23.07	20.40 ± 22.63	19.04 ± 21.24			*
							
	Control	23.80 ± 24.05	24.82 ± 24.56	24.79 ± 85.06	.247	.386	.039

Insomnia	Case	31.29 ± 35.62	21.76 ± 25.04	10.88 ± 18.49	*	.547	*
							
	Control	16.00 ± 27.13	16.66 ± 29.35	17.33 ± 27.13	.001		.001

Appetite loss	Case	7.48 ± 19.56	4.08 ± 12.96	.680 ± 4.76			*
							
	Control	4.08 ± 14.64	5.44 ± 18.44	6.1 ± 21.16	.1840	.711	.005

Constipation	Case	29.93 ± 36.79	30.61 ± 37.16	28.57 ± 35.35	.247	.146	.076
							
	Control	18.05 ± 30.71	20.83 ± 31.97	20.13 ± 32.06			

Dyspnea	Case	7.48 ± 17.03	6.12 ± 14.7	5.42 ± 14.18	.814	.373	.167
							
	Control	8.66 ± 14.76	9.33 ± 16.55	10.00 ± 16.83			

Financial difficulties	Case	36.11 ± 34.26	38.19 ± 35.05	36.80 ± 33.14			
							
	Control	33.33 ± 32.25	34.72 ± 32.94	35.41 ± 33.26	.132	.707	.608

The higher values indicate a greater degree of symptoms, min: 0 max: 100

In comparing the mean score of the functional scale from pre to post and follow up intervention in groups as measured by EORTC QLQ-BR23, the results indicated an increase in all the aspects such as body image, sexual function, sexual satisfaction and future perspective in the experimental group (P < 0.001) and a decrease in all those aspects of life quality in the control group (Table 4)

**Table 4:** Functioning Scores mean of life quality in groups before and after the intervention (as measuredby the EORTC -BR23)

Functioning Scores	Time	Before intervention	After intervention	2 months after intervention	Time	Group	Time/group
			
	Groups	Mean ± SD	Mean ± SD	Mean ± SD			P
Body image	Case	68.19 ± 25.21	82.14 ± 14.29	93.87 ± 6.31	*	*	*
							
	Control	73.33 ± 24.51	72.33 ± 23.35	71.00 ± 23.21	.001	.022	.001

Sexual function	Case	27.13 ± 16.27	43.02 ± 15.09	64.34 ± 13.88	*	*	*
							
	Control	24.63 ± 19.48	23.91 ± 18.80	19.35 ± 22.82	.001	.001	.001

Sexual enjoyment	Case	26.82 ± 18.58	46.34 ± 19.54	76.42 ± 18.62	*	*	*
							
	Control	22.48 ± 22.67	21.70 ± 22.86	20.15 ± 23.16	.001	.001	.001

Future perspective	Case	47.61 ± 32.6	62.58 ± 25.12	88.43 ± 17.41	*	*	*
							
	Control	57.14 ± 35.35	54.42 ± 33.81	51.02 ± 32.70	.001	.040	.001

In comparing the mean score of symptom scale from pre to post and follow up intervention in groups as measured by EORTC QLQ-BR23 in time process, considering the mutual effect of time and group, the results indicated a decrease in symptoms in two aspects of breast symptom (0.032) and upset by hair loss (0.049) in the experimental group 2 months after the intervention and there was an increase in symptoms in the control group 2 months after the intervention (Table 5).

**Table 5:** Means scores of Symptoms of life quality in groups before and after the intervention (as measured by the EORTC-BR23)

Symptom Scores	Time	Before intervention	After intervention	2 months after intervention	Time	Group	Time/group
			
	Groups	Mean ± SD	Mean ± SD	Mean ± SD			P
Systemic therapy side effects	Case	21.82 ± 16.46	12.79 ± 10.63	3.67 ± 4.19	*	.185	*
							
	Control	16.79 ± 18.87	17.04 ± 19.19	17.29 ± 19.69	.001		.001

Breast symptoms	Case	18.87 ± 18.92	18.70 ± 18.90	17.51 ± 19.41			
							
	Control	13.60 ± 15.28	13.94 ± 15.53	14.28 ± 15.77	.404	.209	.032

Arm symptoms	Case	31.97 ± 26.02	31.74 ± 25.15	31.06 ± 24.53			
							
	Control	24.44 ± 18.51	24.88 ± 18.45	24.66 ± 18.55	.532	.120	.246

Upset by hair loss	Case	12.69 ± 19.65	11.11 ± 16.10	9.52 ± 18.68			*
							
	Control	12.28 ± 25.36	14.03 ± 25.61	15.78 ± 25.74	.992	.665	.049

Generally regardless of time there was no significant difference between the two groups regarding those aspects?

## Discussion

The study results show that the peer lead education is an effective approach to improve the life quality of mastectomy patients. This study evaluated the life quality of 99 breast cancer patients using standard life quality assessment tools. The results of the current study indicated an increase in the life quality related to breast cancer before to two months after intervention and support. The outcome of this study verifies the effect of peer lead education on life quality for cancer patients. No significant changes were found in the life quality of the control group. In aspects such as general health, psychological function, social function, mental function and role function increased in the experimental group two months after the intervention. Several reviews have concluded that psychosocial interventions have a positive impact on the well-being of breast cancer patients. To date, research has not established whether one kind of intervention is more effective than another but a variety of intervention types have demonstrated beneficial effects. Many women need to participate in breast cancer support groups to cope with their illness. According to Till (2003), support group and a navigator to support the breast cancer women play an important role in improved quality of life as women need to depend on a source in relation to breast cancer. So assistance in various phases needs to be taken into account in an effort to evaluate the "navigator role" [18]. Although the empiric evidence points to the community-based support groups as beneficial, but other studies have not reported substantial outcomes. In Cook Gotay study on the impact of a peer-Delivered Telephone Intervention on women experiencing a breast cancer recurrence, the results showed that it did not lead to better psychosocial outcomes [19].

In contrast, Liberman's study results (2003) showed participation in the peer education program lead to an improvement in the social-psychological functions in women diagnosed with breast cancer [20]. Also, Cappiello (2007) believe that patients experience many physical disorders as time goes on and they need support and help to cope with their condition [21].

The results of this study reflect a reduction in fatigue, anorexia and insomnia two months after the intervention. In Clark et al.'s study (2003) on the patients who were undergoing radio-therapy there was a reduction in depression, anxiety, loneliness and physical symptoms such as anorexia, gastro-intestinal disorder and fatigue after participating in peer support groups in the experimental group as compared to the control group [22]. The body image, sexual function, satisfaction in sexual performance and attitude towards the future in the experimental group improved two months after the intervention. Ganz et al. (2000) introduce fatigue, anxiety, disorder in the body image, sexual issues and complication in the patients' hand as the most common factors reducing the quality of life in these patients [23]. Hence, the body image, sexual function and satisfaction with the sexual performance are common problems which women experience after mastectomy. In this study the sexual function and satisfaction showed a great change two months after the intervention when compared to before the intervention (p < 0.001), while in Hazrati et al.'s study (2008)there was no improvement in the patients' sexual function [24]. It seems the difference relates to the intervention method. The researchers believe that culture is an inflectional factor in sexual issues and body-image. Also Fobair (2006) believes that Asian women do not like to talk about their sexual issues and consider it shameful and irrational [25]. Therefore, intervention techniques influences women to talk freely about their sexual concerns in groups and find a personal for better sexual function and satisfaction. The results of this study are similar to those of Matthews' research (2002) who states that patients benefit from talking to each other about their sexual issues and generally find more satisfaction in their life [26]. It seems that the peer group method and group counseling is more effective for improving the sexual function because they can talk without shame about their sexual issues in a more relaxed environment.

Regarding the physical function and complications in the breast cancer patient's arm, no significant differences were seen in the experimental group 2 months after the intervention. Lash et al. (2002) in their study showed that the surgical symptoms worsen during the first year after surgery without physiotherapy and breast cancer care [27]. The results of Hazrati et al.'s study (2008) also proves this issue, indicating that these patients need to participate in physiotherapy sessions to improve their physical function and reduce symptoms in their arm and breast as well as participate in the educational sessions. Therefore it seems that participating in physiotherapy sessions alongside peer support is effective [24]. Cadmus et al.'s study (2009) reveals that exercise alone is not effective in breast cancer patients' quality of life, although it improves the social function in treated cancer patients [28]. Therefore lack of improvement in the physical function and the existence of complication in the patients' hand in the experimental group may be the result of not following the care instructions for the hand, not doing organized fitness exercises and not participating in physiotherapy sessions in the peer groups. Generally, it can be concluded that the life quality of the women who participated in peer group education was considerably higher compared to those who did not participate in the sessions. Strong support was reported in Patenaude et al.'s study (2008) from 25 healthy women who had undergone bilateral prophylactic mastectomy and 45 women unilateral prophylactic mastectomy, for the emotional and informational value of speaking with a woman who had previously undergone prophylactic mastectomy [29]. In the current study the majority of participants recognized that peer education was effective and they were willing to participate in all sessions. Therefore, it is recommended that peer education program should be included as part of the patients' treatment program with the aim of reducing the symptoms of cancer and improving the quality of life.

## Conclusions

The results of this study provide empirical evidence about the benefits of incorporating peer-led education in improving the life quality for post-mastectomy patients. It is anticipated our findings will contribute to delegating caring responsibility to these patients and facilitating the establishment of a counseling center in the breast cancer clinics. Overall breast cancer patients greatly benefited from peer group support to improve their quality of life.

## Abbreviations

EORTC QLQ-C30: European Organization for Research and Treatment of Cancer Quality Questionnaire-Cancer 30; EORTC QLQ-BR23: European Organization for Research and Treatment of Cancer Quality of Life Breast Cancer Questionnaire.

## Competing interests

The authors declare that they have no competing interests.

## Authors' contributions

FSH was the main investigator, coordinating the research and writing the paper. NA was responsible for data collection and contributed to the data analysis. MH assisted in the study design and coordinated the research. ST helped for interviewing the patients and introduced them for intervention. NZ did the data analysis and give statistical advice. All the authors read and approved the final manuscript.
